# Perceptions of AI‐generated informational texts and effects of the source: An online experiment

**DOI:** 10.1111/aphw.70223

**Published:** 2026-10-06

**Authors:** Laura M. König, Maren C. Podszun, Wolfgang Gaissmaier, Helge Giese

**Affiliations:** ^1^ Faculty of Psychology University of Vienna Vienna Austria; ^2^ Institute of Nutritional Sciences University of Hohenheim Stuttgart Germany; ^3^ Department of Psychology University of Konstanz Konstanz Germany; ^4^ Heisenberg Chair for Medical Risk Literacy and Evidence‐based Decisions Charité Universitätsmedizin Berlin Berlin Germany

**Keywords:** ChatGPT, generative AI, large language models, nutrition communication, public health nutrition

## Abstract

Artificial intelligence (AI) is becoming increasingly common for health information seeking, yet many people are still skeptical of its performance. This between‐subjects online experiment tested whether informing readers that a text was produced by AI influenced their ratings in 774 participants. They were randomly assigned to read one of six brief texts and rated trustworthiness, interest, and perceived understanding, as well as intention to act upon the received information and willingness to share the information with others. Source information did not influence ratings, but only 41.5% of participants in the AI source condition noticed the source. Source awareness was associated with higher education and greater nutrition knowledge. Nutrition information produced by AI chatbots is generally seen as trustworthy, although source information is often ignored. Our research highlights critical implications for public health messaging in the digital age as consumers may not differentiate between information generated by AI versus other sources, although future research is required to assess source perceptions in AI‐generated texts in more detail.

## INTRODUCTION

Since the release of ChatGPT in 2022, artificial intelligence (AI) has become increasingly accessible to the general public. The increased availability of AI chatbots is also mirrored in increased use of the technology in various areas of life. In the US, around 40% of the population have experience in using generative AI (Bick et al., 2025, February 11); in the UK, estimates are slightly higher (Office for National Statistics, 2023), and in Taiwan, over 70% of the population had used ChatGPT before (Greussing et al., 2025). The majority of prompts generated by these users focused on (creative) writing tasks and role play (21%), followed by homework help (18%), and search and other inquiries (17%); another 5% of queries focused on health and advice (Merrill & Lerman, 2024; Zhao et al., 2024). Although yet a relatively small number in comparison to other use cases, it still indicates that lay people are already turning to AI chatbots with questions about their health. Given the general public interest in obtaining health‐related information online (Eurostat, 2022; Wang & Cohen, 2023), it can be expected that the interest in also using AI for this purpose—and therefore its importance for health communication—will only grow further. AI chatbots thus will become increasingly important for public health, where they have the potential to provide real‐time health information, including lay people‐appropriate summaries of guidelines and scientific findings, and refute misinformation (Panteli et al., 2025).

### AI‐generated nutrition advice

Indeed, AI has demonstrated its usefulness in many health‐related domains. For example, it is already outperforming human experts in several diagnostic tasks, including brain tumour (Khalighi et al., 2024) and skin cancer diagnoses, at least when compared to medical generalists (Salinas et al., 2024). However, health advice obtained by AI chatbots seems to be somewhat less accurate, for example, when it comes to advice for specific diagnoses such as ophthalmic diseases (Cappellani et al., 2024) or medical fields such as urology (Whiles et al., 2023), as well as nutritional advice for patients with various conditions (Ponzo et al., 2024) and meal plans for healthy individuals (Hieronimus et al., 2024). This indicates that, currently, advice provided by AI chatbots still may need to be taken with a grain of salt, although the algorithms and their output are steadily improving (Rajpurkar et al., 2022).

The public seems to be aware of potential shortcomings of AI chatbots, as is indicated by only medium levels of trust in AI (Link & Beckmann, 2025) and lower reliability evaluations and willingness to follow the provided advice if indicated that AI was involved in its generation (Reis et al., 2024), which may explain the relatively low use rates of AI chatbots for this purpose. Other studies, however, indicate higher levels of trust in AI‐generated advice and similar levels compared to human‐generated advice (Huschens et al., 2023). The latter study also indicates that AI‐generated texts may be more accessible and engaging than texts generated by humans (Huschens et al., 2023), which is an important prerequisite for understanding of, interest in, and finally acceptance of (scientific) information and action (Friedrich & Heise, 2022; König et al., 2025; Spencer et al., 2019). Given the sparse and inconsistent findings, the present study therefore experimentally tested whether indicating that an informational text about nutrition was generated by AI influenced trustworthiness, interest and perceived understanding as core evaluation criteria of informational texts, as well as intention to act in line with the received information and willingness to share the information with others as relevant potential outcomes of this evaluation.

### Factors influencing technology evaluation

The evaluation of any technology is, among others, influenced by prior experience: novel technologies are often perceived as riskier because they are unknown (Slovic et al., 1980). This fact may also explain the divergence in the aforementioned findings: Three quarters of the sample in Huschens et al. (2023) reported having used large language models, which lie behind AI chatbots, at least once. The other two studies that reported lower trust ratings did not report on the samples' familiarity with AI (Link & Beckmann, 2025; Reis et al., 2024), although Reis et al. (2024) partly report on a nationally representative sample from the UK, where about half of the population has experience with using AI (Office for National Statistics, 2023). Nonetheless, taking prior experience into account could be crucial to better understand whether reservations might be reduced through mere exposure (Zajonc, 1968).

Moreover, not everyone may pay attention to the source of online information. For instance, individual differences in motivation and interest for a topic may influence whether source information is obtained (Paul et al., 2017). In the context of nutrition advice, nutrition knowledge and adhering to a healthy diet could be proxies for this motivation and interest. Paying attention to the source of online information also is an aspect of digital (health) literacy, which is associated with several sociodemographic characteristics. Specifically, younger age, higher education level, and income are positively associated with digital health literacy (Estrela et al., 2023). Considering these sociodemographic as well as psychological and behavioral correlates thus seems important when evaluating whether source information is appraised.

### The present study

Based on initial evidence derived from the available literature, this study explored the following research questions (RQ). First, we tested whether texts flagged as produced by AI are rated differently regarding trustworthiness, interest, perceived understanding, intention to act upon the received information, and willingness to share the information with others, compared to texts where no source is provided (RQ 1). Second, we investigated whether these relationships were moderated by prior experience with using AI to obtain nutrition‐related information and prior experience with using AI in general (RQ 2). Third, we examined whether certain sociodemographic (age, gender, location, education, income, employment), anthropometric (body mass index [BMI] as a characteristic linked to diet), psychological (affinity for technology, nutrition knowledge), or behavioral (healthy diet, as measured by the Healthy Eating Index) characteristics were related to individuals' awareness of the source, measured by them explicitly identifying AI as the source of the text (RQ 3).

## MATERIALS AND METHODS

This study was preregistered on the Open Science Framework (OSF) prior to data collection (König et al., 2024; please see https://osf.io/mexj9). This study was conducted according to the preregistration unless mentioned otherwise in the respective sections of this manuscript. Materials (German original and English translation), data, and code are available from the project's OSF page (https://osf.io/t5arq/). General ethical approval was obtained from the University of Konstanz ethics committee.

### Sample

An ISO 20252‐certified panel provider was used to recruit a sample of *N* = 828 adults (see Table S1) that was representative of the German population regarding age, gender, and level of education (see König et al., 2024). The panel provider uses an elaborate strategy to prevent bot responses, including real‐time checks at initial registration for the panel such as IP checks and captchas, quality control of profile information and responses in surveys, and ongoing checks of IT infrastructure.

The sample size was based on an a priori power calculation in G*Power 3.1 (Faul et al., 2009) for a two‐tailed independent samples *t*‐tests to test the source effects postulated in RQ 1 with 1 − *β* = 0.8, *α* = 0.05, and Cohen's *d* = 0.2 as smallest effect size of interest, which represents a small effect according to Cohen (1992). We furthermore accounted for 5% dropout because of a seriousness check.

### Design and procedure

The data reported in the present analysis were collected as part of a larger study on AI and nutrition‐ and health‐related information. The part of the survey used in the present analysis applied a 2 source (AI vs. no source) × 3 topic (diet, supplements, snacks) between‐subjects design. We decided to include three different topics to test the generalizability of the findings; to test whether results may differ by text, topic was also included as a control variable in the analyses.

Upon accessing the survey, participants read the study description, which informed them that this study was about nutrition‐ and health‐related information and perceived quality of related informational texts. They then provided informed consent by ticking a box. They then provided sociodemographic and anthropometric information, indicated their affinity for technology, and reported on their current eating habits and nutrition knowledge. They were then randomly assigned to one of the six experimental conditions using the random algorithm built into the survey tool Tivian Unipark. Afterwards, they rated the text regarding trustworthiness, interest, perceived understanding, intention to act upon the received information, and willingness to share the received information with others. They were also instructed to draft a WhatsApp message to a friend retelling the received information. Afterwards, they were explicitly asked whether a source was indicated in the text they just read, which acted as the manipulation check. The participants were then informed that the text was produced by ChatGPT. Finally, they reported whether they previously used AI to obtain nutrition‐ or health‐related information or used AI for other purposes, provided further details on such use where applicable, and reported their attitudes toward using AI to obtain nutrition‐ and health‐related information and their intentions to use AI for these purposes in the future. These measures are reported elsewhere (König et al., 2025, April 24). Finally, the participants were debriefed and redirected to the panel provider for payment.

### Materials and measures

The questionnaire and texts used in this study can be found in the Supporting Information.

#### Seriousness check

At the end of the survey, the participants were asked whether they think their data should be used for analysis (yes/no).

#### Experimental manipulation

Three brief texts were created with the free version of ChatGPT on 30 September 2024. Three texts were generated to test whether the effects were consistent across multiple prompts and topics. The texts are responses to brief prompts based on common questions dieticians receive about nutrition (Kirk et al., 2023), which were individually entered into ChatGPT. Afterwards, the texts were checked for correctness by a member of the study team who is a nutritional scientist and used as‐is, that is, without any corrections to the content or language or modification of the length. Without further information about the source, the texts were used as the “no source” control. In the AI condition, the following sentence was added immediately below the text: “Note: This text was written by ChatGPT, an artificial intelligence.” Without this add‐on, the length of texts including the prompt range from 109 to 190 words.

#### Manipulation check

The participants were asked whether it was mentioned who wrote the text (yes/no); if yes, the participants are asked to indicate the source.

##### Trustworthiness

*Trustworthiness* of the text was assessed with four 7‐point semantic differentials: not at all trustworthy versus completely trustworthy; very dishonest versus very honest; very dubious versus very reputable; very implausible versus very credible (Giese et al., 2021). The mean of the four items was calculated (Cronbach's *α* = 0.92).

##### Interest

*Interest in the text* was assessed with one item, asking the participants to indicate on a 7‐point semantic differential whether they found the text (1) very boring to (7) very captivating.

##### Perceived comprehension

*Perceived comprehension* was assessed with a 7‐point semantic differential ranging from 1 (*very incomprehensible*) to 7 (*very comprehensible*) (Kotz et al., 2023).

##### Intention

*Intention* to act upon the information received in the text was assessed with one item on a 7‐point scale ranging from 1 (*no, certainly not*) to 7 (*yes, in any case*).

##### Willingness to share

*Willingness to share* the information with others, either while talking in person or on social media, was assessed with two items on a 7‐point scale from 1 (*no, certainly not*) to 7 (yes, in any case) (c.f., Giese et al., 2021; Kotz et al., 2023). The mean of the two items was calculated (Cronbach's *α* = 0.74).

#### Information shared with others

The participants were asked to draft a WhatsApp message to a friend summarizing the information they just received in an open text field. Diverging from the preregistration, we coded the data into AI mentioned as source (1), AI not mentioned as source (0), and missing value if the messages were a nonsensical combination of characters (e.g. “sdf”).

#### Use of AI to obtain nutrition‐ and health‐related information

The participants were asked whether they previously used AI to obtain nutrition‐ or health‐related information or for other purposes (yes/no).

##### Affinity to technology

*Affinity for technology* was assessed with the short‐form scale (Wessel et al., 2019). Items 3 and 4 were inverse‐coded before calculating the mean of the four items.

#### Healthy Eating Index

The participants completed the Food Frequency Questionnaire by Winkler and Döring (1995, 1998), based on which we calculated the Health Eating Index (HEI) following the instructions provided by the authors in Winkler and Döring (1995).

##### Nutrition knowledge

Nutrition knowledge was assessed with the German version (Koch et al., 2021) of the Consumer‐oriented Nutrition Knowledge Scale (CoNKS) (Dickson‐Spillmann & Siegrist, 2011). Responses were categorized as correct or false based on the information provided by the authors, and the number of correct responses was determined as the score.

### Data analysis

If not otherwise stated, the data were analyzed as preregistered (König et al., 2024).

#### Data cleaning

Diverging from the preregistration, we had to remove values for height and weight for five participants because they indicated implausible values for height or weight.

#### Missing data

For the quantitative variables, there was no missing data. For the open text fields, 2.3% of responses were missing for the WhatsApp message, and no responses were missing for obtaining health‐related information or using AI for other purposes. However, 17.7% of responses for obtaining health‐related information and 9.5% per cent of responses for using AI for other purposes were nonsensical (e.g. “Hgh”) and were thus deleted.

#### Descriptive statistics

Descriptive statistics (age, gender, residency, years of education, monthly household budget, employment, BMI) were calculated for the full sample and per group and compared using between‐subjects analyses of variance (ANOVAs) and Chi‐square tests as appropriate. During the revision process, we were asked to remove the preregistered comparisons of sociodemographic information between conditions. The results of this test are available from the study's OSF page. In addition, although not preregistered, we also report the proportion of participants who reported prior experience of using AI to obtain nutrition‐related information and using AI in general.

#### Manipulation check

A Chi‐square test was conducted to test for differences between the AI and no source conditions regarding the frequency of indicating that a source was listed. Cramer V was reported as the effect size recommended in Goss‐Sampson (2022) and Jané et al. (2024).

#### Test of research questions 1 and 2

Effects of the source (RQ 1) were tested using independent samples *t*‐tests and 2 *Source* × 3 *Topic* between‐subjects ANOVAs with the outcome variables trustworthiness, interest in the text, perceived comprehension, intention, and willingness to share. Topic was included as a between‐subjects factor to test for potential differences between texts via a Source by Topic interaction. As preregistered, we only followed up the significant effect for trustworthiness to test for potential moderating effects of prior experience with AI both in general and for obtaining nutrition‐related information (c.f. RQ 2), which was only the case in the full sample, but not for the subsample that indicated that their data should be used for research. Because only 41.5% of participants in the AI conditions correctly identified the source, we additionally explored whether this awareness of the AI source influenced the results by additionally conducting 3 *Source Awareness (yes/no/no source provided)* × 3 *Topic* between‐subjects ANOVAs with the outcome variables trustworthiness, interest in the text, perceived comprehension, intention, and willingness to share; this additional analysis was not preregistered.

#### Test of research question 3

We tested for when individuals correctly identified the source (RQ 3); diverging from the preregistration, the logistic regression testing the effects of sociodemographic (age, gender, residency, education, income, occupation) and anthropometric (BMI) factors, affinity for technology, nutrition knowledge, and HEI was conducted only for the manipulation check variable and only among participants assigned to the AI source condition.

#### Inference criteria

In line with the preregistration, alpha was set to 0.05 to determine statistical significance in all analyses. Because statistical significance is not necessarily equal to practical significance (Peeters, 2016), especially when using relatively large samples as in the present study, we also report effect sizes and interpret them according to Cohen (1992), as preregistered. Finally, because these effect sizes are only point estimates, we added confidence intervals as suggested during the revision process.

## RESULTS

### Description of the sample

The sample comprised 828 adult participants, out of which *n* = 54 (6.5%) indicated that their data should not be used for analysis in the seriousness check. Because this was above the predetermined threshold of 5% in the preregistration, we additionally report analyses using the full sample in the Supporting Information (including Tables S2–S7). In the following, we report analyses using the *N* = 774 participants who indicated that their data could be used. Sociodemographic characteristics for these 774 participants and breakdown by condition are reported in Table 1.

**TABLE 1:** Sociodemographic characteristics of the full sample and by condition.

		Full sample (*N* = 774)	By condition					
			AI: no topic: diets (*n* = 129)	AI: no topic: supplements (*n* = 131)	AI: no topic: snacks (*n* = 131)	AI: yes topic: diets (*n* = 127)	AI: yes topic: supplements (*n* = 131)	AI: yes topic: snacks (*n* = 125)
Age (*M*, *SD*)		42.44, 13.21	43.36, 12.16	40.97, 13.39	42.04, 12.97	42.55, 14.32	42.91, 12.41	42.86, 13.70
Gender^a^	Women	51.0%	48.8%	48.1%	48.9%	44.1%	55.0%	61.6%
	Men	49.0%	51.9%	51.9%	51.1%	55.9%	45.0%	38.4%
	Diverse	0.0%	0.0%	0.0%	0.0%	0.0%	0.0%	0.0%
Residency	City with more than 100,000 inhabitants	36.6%	35.7%	40.5%	29.0%	27.6%	45.8%	40.8%
	City with between 20,000 and 100,000 inhabitants	26.0%	24.8%	26.0%	36.6%	27.6%	22.9%	17.6%
	Town with between 5000 and 20,000 inhabitants	18.5%	20.2%	16.0%	14.5%	19.7%	16.0%	24.8%
	Village with up to 5000 inhabitants	19.0%	19.4%	17.6%	19.8%	25.2%	15.3%	16.8%
Years of education (*M*, *SD*)		14.02, 3.10	14.10, 3.20	14.33, 3.22	13.57, 3.11	14.10, 3.18	14.18, 2.85	13.86, 3.02
Monthly household budget		Median = 2500€–3000€ Interquartile range from 1500€–2000€ to 3000€–5000€	Median = 2500€–3000€ Interquartile range from 1500€–2000€ to 3000€–5000€	Median = 2500€–3000€ Interquartile range from 1500€–2000€ to 3000€–5000€	Median = 3000€–5000 € Interquartile range from 2500€–3000€ to 3000€–5000€	Median = 2500€–3000€ Interquartile range from 1500€–2000€ to 3000€–5000€	Median = 2500€–3000€ Interquartile range from 1500€–2000€ to 3000€–5000€	Median = 2500€–3000€ Interquartile range from 1000€–1500€ to 3000€–5000€
Employment	Full‐time employment	52.7%	56.6%	51.9%	57.3%	52.8%	48.9%	48.8%
	Part‐time employment	21.1%	22.5%	18.3%	16.0%	22.0%	29.0%	18.4%
	Job‐seeking	7.4%	5.4%	7.6%	8.4%	8.7%	5.3%	8.8%
	In training	5.4%	3.1%	8.4%	6.1%	5.5%	2.3%	7.2%
	Homemaker	6.5%	6.2%	9.2%	6.9%	3.9%	7.6%	4.8%
	Retired	7.0%	6.2%	4.6%	5.3%	7.1%	6.9%	12.0%
BMI (*M*, *SD*)		26.99, 6.33	26.67, 6.03	26.91, 5.95	26.88, 6.84	26.75, 6.13	27.01, 6.20	27.76, 6.85
Prior experience with AI for obtaining nutrition‐related information	Yes	31.3%	34.1%	33.6%	32.8%	26.8%	31.3%	28.8%
	No	68.7%	65.9%	66.4%	67.2%	73.2%	68.7%	71.2%
Prior experience with AI in general (% yes)	Yes	37.5%	34.1%	41.2%	35.1%	32.3%	36.6%	45.6%
	No	62.5%	65.9%	58.8%	64.9%	67.7%	63.4%	54.4%

^a^ No participant indicated diverse gender.

### Manipulation check

The Chi‐square test indicated that the participants differed in indicating a source depending on the AI condition (AI vs. no source). In the no source conditions, 93.1% of the participants indicated that no source was provided, whereas only 58.5% in the AI conditions indicated that no source was provided, *χ* ^2^(*df* = 1) = 126.94, *p* < .001, *Cramer V* = 0.41. Given the large number of participants who were unable to remember the source, the manipulation was unsuccessful in a substantial proportion of the sample; analyses were thus reported as planned and by comparing participants aware and unaware of the source label, and also taking source awareness into account.

From the 159 participants in the AI condition who indicated that a source was provided, 135 participants (84.9%) had correctly identified AI, either by directly indicating ChatGPT (*n* = 80) or an AI more generally (*n* = 55). The remaining participants had given nonsensical responses (e.g. “erdfhb”; *n* = 10) indicated to not remember or to not know (*n* = 9), left the text box empty (*n* = 4), or indicated a university (*n* = 1).

### Are texts marked as produced by AI perceived differently compared to texts where no source is indicated?

First, we conducted independent samples *t*‐tests to test for the effects of indicating that a text was produced by AI on trustworthiness, interest, perceived understanding, intention to act upon the information received, and willingness to share the received information with others. For all comparisons, *p*‐values were greater than .05 with very small effect sizes (Cohen's *d* ranging from −0.03 to 0.14; see Table 2). To test for potential differences between topics, we also conducted 2 *Source* × 3 *Topic* between‐subjects ANOVAs with the same outcomes (see Table 3).

**TABLE 2:** Results of independent samples *t*‐tests comparing ratings between AI and no source conditions.

Outcome	AI condition (*M, SD*), *n* = 383	No source condition (*M, SD*), *n* = 391	*t*	*df*	*p*	Cohen's *d*		
						Point estimate	95% CI, lower bound	95% CI, lower bound
Trustworthiness	5.21, 1.25	5.38, 1.28	1.88	772	.061	0.14	−0.01	0.28
Interest	4.66, 1.49	4.76, 1.60	0.91	772	.362	0.07	−0.08	0.21
Perceived understanding	5.61, 1.44	5.66, 1.46	0.52	772	.604	0.04	−0.10	0.18
Intention	4.38, 1.56	4.47, 1.62	0.83	772	.409	0.06	−0.08	0.20
Willingness to share	3.65, 1.60	3.61, 1.66	−0.34	772	.731	−0.03	−0.17	0.12

**TABLE 3:** Results of between‐subjects ANOVAs comparing ratings between conditions.

Outcome	AI: no			AI: yes			AI source (*df*s 1, 768)			Topic (*df*s 2, 768)			AI source * topic (*df*s 2, 768)		
	Topic: diets (*n* = 129)	Topic: supplements (*n* = 131)	Topic: snacks (*n* = 131)	Topic: diets (*n* = 127)	Topic: supplements (*n* = 131)	Topic: snacks (*n* = 125)	*F*	*p*	Partial *η* ^2^	*F*	*p*	Partial *η* ^2^	*F*	*p*	Partial *η* ^2^
Trustworthiness	5.48 (1.25)	5.40 (1.31)	5.26 (1.28)	5.01 (1.24)	5.31 (1.22)	5.31 (1.27)	5.68	.059	0.01	0.52	.596	<0.01	2.94	.054	0.01
Interest	5.00 (1.54)	4.67 (1.68)	4.62 (1.58)	4.54 (1.44)	4.81 (1.50)	4.63 (1.53)	0.89	.347	<0.01	0.62	.541	<0.01	2.73	.066	0.01
Perceived understanding	5.67 (1.54)	5.59 (1.51)	5.74 (1.33)	5.36 (1.45)	5.66 (1.46)	5.81 (1.38)	0.27	.607	<0.01	2.08	.126	0.01	1.45	.235	<0.01
Intention	4.41 (1.69)	4.50 (1.70)	4.50 (1.49)	4.09 (1.41)	4.51 (1.41)	4.52 (1.66)	0.69	.401	<0.01	2.20	.111	0.01	0.93	.394	<0.01
Willingness to share	3.71 (1.62)	3.61 (1.68)	3.53 (1.66)	3.53 (1.53)	3.83 (1.49)	3.60 (1.78)	0.10	.748	<0.01	0.61	.542	<0.01	0.99	.373	<0.01

### Are the influences of AI moderated by prior experience with using AI to retrieve nutrition‐related information or prior experience with using AI in general?

We preregistered that we would only test for moderating effects if the main effects tested in RQ 1 were statistically significant. Because there were no statistically significant main effects, we did not conduct these analyses.

### Additional analyses taking source awareness into account

Because only 41.5% of participants correctly identified an AI source in the AI source condition, we conducted additional 3 *Source Awareness (source condition + aware/source condition + not aware/no source condition)* × 3 *Topic* between‐subjects ANOVAs to test whether source awareness might have influenced the results for the AI source conditions. Results are summarised in Table 4. In brief, for the outcomes trustworthiness, interest in the text, and perceived comprehension, *p*‐values were greater than .05 (partial *η* ^2^s ≤ 0.01). For intention, there was a main effect of topic, *F*(2, 765) = 3.47, *p* = .032, partial *η* ^2^ = .01, but *p*‐values for the Bonferroni‐corrected post‐hoc tests were greater than .05. For willingness to share, there was a main effect of source awareness, *F*(2, 765) = 4.05, *p* = .018, partial *η* ^2^ = 0.01. The participants in the AI conditions who were aware of the AI source were somewhat less likely to share the information with others (*M* = 3.34, *SD* = 1.53, 95% CI [3.06; 3.61]) than the participants in the AI conditions who were unaware of the AI source (*M* = 3.83, *SD* = 1.61, 95% CI [3.63; 4.03]; *p* = .014).

**TABLE 4:** Results of between‐subjects ANOVAs comparing ratings between conditions, taking into account awareness of the AI source in the AI source conditions.

	AI source (*df*s 2, 765)			Topic (*df*s 2, 765)			AI source * topic (*df*s 4, 765)		
Outcome	*F*	*p*	Partial *η* ^2^	*F*	*p*	Partial *η* ^2^	*F*	*p*	Partial *η* ^2^
Trustworthiness	2.12	.121	0.01	1.27	.283	<0.01	1.53	.192	0.01
Interest	1.36	.256	<0.01	0.48	.622	<0.01	1.47	.209	0.01
Perceived understanding	2.49	.084	0.01	2.16	.116	0.01	0.89	.470	0.01
Intention	2.57	.077	0.01	2.47	.03	0.01	0.67	.602	<0.01
Willingness to share	4.05	.018	0.01	0.93	.395	<0.01	.085	.492	<0.01

In line with the preregistration, we followed up the latter finding with a linear regression, testing for potential moderation effects by prior experience with AI both in general and for obtaining nutrition‐related information, which yielded no statistically significant results (see Table 5).

**TABLE 5:** Results of the linear regression testing for the effects of the source and prior experience with AI on the willingness to share the information with others, taking into account awareness of the AI source in the AI source conditions.

Predictor	*b*	SE(*b*)	95% CI for b		*β*	*t*	*p*
			Lower bound	Upper bound			
Constant	3.29	0.11	3.07	3.51		29.39	<.001
AI source: no source (0) vs. aware of indicated source (1)	−0.31	0.23	−0.76	0.13	−0.07	−1.39	.166
AI source: no source (0) vs. unaware of indicated source (1)	0.25	0.18	−0.09	0.60	0.07	1.44	.149
Prior experience with AI to obtain nutrition‐related information	0.41	0.25	0.07	0.90	0.12	1.68	.094
Prior experience with AI in general	0.38	0.23	−0.08	0.83	0.17	1.62	.106
AI source: no source (0) vs. aware of indicated source (1) * prior experience with AI to obtain nutrition‐related information	0.60	0.45	−0.29	1.49	0.08	1.33	.184
AI source: no source (0) vs, unaware of indicated source (1) * prior experience with AI to obtain nutrition‐related information	−0.08	0.40	−0.88	0.71	−0.02	−0.20	.840
AI source: no source (0) vs. aware of indicated source (1) * prior experience with AI in general	−0.20	0.41	−0.99	0.60	−0.04	−0.48	.628
AI source: no source (0) vs. unaware of indicated source (1) * prior experience with AI in general	0.02	0.38	−0.72	0.76	0.01	0.06	.950

*Note*: Prior experience with AI to obtain nutrition‐related information coding 0 = no prior experience, 1 = prior experience; prior experience with AI coding 0 = no prior experience, 1 = prior experience.

### Do individuals explicitly mention if a text has been produced by AI when communicating about the information with others? Are certain sociodemographic, psychological, or behavioral characteristics associated with the likelihood of mentioning AI as the source?

#### Results based on the WhatsApp message item

A substantial number of participants refused to write a message (15.8%), for example, because they did not use WhatsApp or did not know what to write. Another 6.3% of the participants left the box empty or used indicators such as “...” to signal that they would prefer not responding. Finally, 4.4% of participants gave a nonsensical response. From the 569 participants (73.5% of the sample) who wrote a message, only 17 (i.e. 3.0%) mentioned AI as the message source; two of these participants explicitly indicated that they refused to write a message because they do not trust information provided by AI, and two more indicated doubts about the validity of the information in their message. We therefore decided against conducting the planned analysis using the coded data as the outcome.

#### Results based on the manipulation check item

In the AI condition, 41.5% per cent (i.e. *n* = 159 out of *N* = 383 in this condition) of the participants indicated that a source had been mentioned, whereas only 6.9% in the no source condition had indicated a source. As indicated earlier, of the 159 participants in the AI conditions who indicated that a source had been given, 135 (84.9%) correctly identified ChatGPT or an AI as the source. In the no source condition, three participants indicated a university or scientific text to have been the source, one listed Facebook, and four named a friend or an individual they know or themselves (potentially because of confusion with the preceding WhatsApp exercise); seven indicated not knowing or not remembering; and two said that they did not pay attention. Seven responses were nonsensical, and four were missing. A logistic regression conducted among the participants in the AI conditions (see also Table 6) indicated that the likelihood of correctly identifying AI as the source was higher in participants with more years of education (*p* = .004, OR = 1.14, 95% CI [1.04; 1.25]) and with higher nutrition knowledge (*p* < .001, OR = 1.15, 95% CI [1.07; 1.23]).

**TABLE 6:** Results of logistic regression conducted with *N* = 383 participants in the AI conditions testing for effects of sociodemographic, psychological, and behavioral characteristics on having correctly identified AI as the source (*n* = 135).

	*b*	SE(*b*)	Wald (*df* = 1)	*p*	OR	95% CI for OR	
						Lower bound	Upper bound
Constant	−4.17	1.01	16.04	<.001	0.02		
Gender^a^	0.28	0.26	1.18	.278	1.32	0.80	2.20
Residency^b^							
Medium‐sized city	−0.12	0.32	0.14	.706	0.89	0.47	1.66
Small city	0.19	0.32	0.35	.555	1.21	0.64	2.27
Village	0.12	0.33	0.13	.718	1.13	0.59	2.17
Income^c^							
Second quartile	0.47	0.38	1.52	.218	1.60	0.76	3.36
Third quartile	0.31	0.37	0.70	.403	1.36	0.66	2.80
Fourth quartile	0.35	0.43	0.67	.413	1.42	0.62	3.26
Employment^d^							
Part‐time	0.05	0.31	0.02	.881	1.05	0.57	1.92
Job‐seeking	0.84	0.50	2.91	.088	2.33	0.88	6.13
In training	0.41	0.57	0.53	.466	1.51	0.50	4.62
Homemaker	−0.24	0.60	0.16	.690	0.79	0.24	2.55
Retired	−0.05	0.49	0.10	.921	0.95	0.37	2.48
Age	0.00	0.01	0.07	.785	1.00	9.98	1.02
Education	0.13	0.05	8.29	.004	1.14	1.04	1.25
ATI	−0.05	0.11	0.18	.670	0.95	0.77	1.19
HEI	−0.50	0.03	2.37	.124	0.95	0.89	1.01
CoNKS	0.14	0.04	14.68	<.001	1.15	1.07	1.23

Abbreviation: CoNKs Consumer‐oriented Nutrition Knowledge Scale.

^a^ Comparator: women.

^b^ Comparator: big city.

^c^ Comparator: lowest quartile.

^d^ Comparator: full‐time employment.

### Results in the full sample

The results remained very similar when also considering the participants who indicated that their data should not be used for analysis (see Supporting Information); the only exception is a very small effect of source on trustworthiness (*p* = .030; Cohen's *d* = 0.15; Cohen, 1992). When we followed up this effect to test for a potential moderation, all *p*‐values were greater than .05.

## DISCUSSION

In this study, we tested whether flagging a text as generated by AI affects its perception and evaluation. Overall, we find that more than 58% of a representative German sample ignored this note and—even if recognized—did not devalue a flagged message, irrespective of prior experience with AI. Consequently, only a negligible minority included the AI source information in their communications, and then only to raise doubts about the validity of the flagged information. The ones that paid attention to the AI source information were more likely to be more knowledgeable about nutrition and of higher education. These findings provide some insights into how AI‐generated information is treated by the public.

### Source neglect

The fact that the source information was practically ignored by a majority of readers raises doubts on whether warning labels that a content is AI generated are sufficient to help readers contextualize the respective message. This becomes even more apparent when one considers that the flag is typically lost in further social interactions. Although one could use visual highlights to make the flagging more notable, this may not meaningfully improve the quality of the conveyed information given that only 3% of the participants that noticed the flag decided to include this information in their own messages. These findings are in line both with a small number of studies comparing AI‐generated to human‐generated texts (Gilardi et al., 2024; Huschens et al., 2023) and with a more general notion for the disregard of source information as indicator for message quality (Aslett et al., 2022; Dias et al., 2020), suggesting that AI source information may fail to help readers interpret a message. This is somewhat at odds with the literature on source effects in science communication that indicated that expert sources lead to higher perceived quality and trust (König et al., 2025; König & Breves, 2021). Indeed, the present study did not contain an expert label condition, which has been shown to lead to more positive evaluations than an AI label or no source information in a recent study on vaccinations (Beckmann et al., 2025). This underlines the need for better understanding both lay views on how AI integrates evidence, especially because several biases of generative AI, based on the input data, are well documented (Rajpurkar et al., 2022), and source attribution if no source is explicitly provided.

Importantly, whether readers pay attention to a text's source may also depend on their representation of the task at hand. This is, for instance, reflected in the RESOLV model (Rouet et al., 2017), which postulates that the physical and social context of reading, including requests from other individuals, influence which features will be extracted from a text. In the present study, no information was given to the participants regarding tasks as part of this study; they were only informed that they would be asked questions about the text after reading it. To some participants, this may have implied a focus on the content rather than on the source. Future work thus may need to be more explicit about subsequent tasks to potentially reduce confounding effects of source neglect. Notably, source information may only be verified if readers encounter discrepant information (e.g. in multiple texts); because participants only read one brief text in the present study, this lack of divergent information may have further contributed to the source neglect (c.f. Discrepancy‐Induced Source Comprehension [D‐ISC] Model, Braasch & Bråten, 2017).

In line with prior research on digital health literacy in general as well as some studies on individual differences in sourcing identified in a recent systematic review (Anmarkrud et al., 2022), the participants with more years of education were more likely to remember the source correctly (Estrela et al., 2023; Salmerón et al., 2018). This may be because they are more aware of the importance of verifying the source, which is considered an advanced reading skill (Salmerón et al., 2018). Educational measures are thus required to improve information literacy in the population to ultimately reduce the spread of false information (Addy, 2020). Moreover, and also in line with a previous systematic review on individual differences in sourcing (Anmarkrud et al., 2022), the participants with better nutrition knowledge were more likely to correctly remember the source. This could be because they may spend more time researching nutrition information and thus are aware of the importance of checking the source because of many false claims about nutrition being spread in various contexts, including online communication (Denniss et al., 2023). Alternatively, their higher level of knowledge about the topic may also free working memory resources that can be spent on identifying the source information (Rouet et al., 2017). Furthermore, if the motivation to learn more about the content is lacking, as could be indicated by low levels of knowledge, individuals are less likely to look for source information because they want to achieve the goal with the least possible effort (Paul et al., 2017). Monetary incentives might be effective in increasing validation efforts, yet they are difficult to implement in real‐world information search scenarios (Panizza et al., 2021).

### Trust in AI‐generated nutrition advice

Instead of the source, readers appear to prioritize other cues in a message to scrutinize its qualities, as negative effects of the AI flag on the social diffusion of the message and its trustworthiness are negligible. Importantly, trustworthiness of the presented information was generally high (means of over 5 on a 7‐point scale). Descriptively, this level of trust was higher than trust in AI news articles reported in prior research, which indicated a mean of 4.4, also using a 7‐point scale from one to seven (Morosoli et al., 2024). This may suggest that the participants used other indicators than source information for their evaluation of the texts' quality, such as their being generally well‐structured and written in a clear language, which are cues that may foster trust compared to some human‐generated information (Huschens et al., 2023; Toma & D'Angelo, 2015). In addition, they were reviewed by a nutritional scientist to ensure they were in line with the current scientific evidence and recommendations by national public health authorities, so could rightfully be considered trustworthy. Still, as AI chatbots may further mimic human communication and optimize the language to generate trust (e.g. Cohen et al., 2024), readers may increasingly rely on invalid cues for trusting a message which is especially concerning when the message provides health information.

Similarly, to the current date it remains unclear whether an AI flag as a cue should be considered a warning sign for low‐validity information, or rather a seal of high quality. With the increased public scrutiny of AI, people may learn for which situations AI can be considered a good counsellor and for which tasks it should be avoided (Qin et al., 2025). This relationship may be further complicated by quality updates of the AI and differences between the emerging AI generators in use (Hieronimus et al., 2024). In this line of reasoning, the prior experience with AI assessed in the current sample was apparently insufficient to systematically help decide whether AI‐generated information can be trusted. We only asked the participants to report whether they had ever used AI to obtain nutrition‐related information or used AI in general, but did not assess frequency of use. Thus, many participants may only have tried out an AI chatbot once (c.f. Huschens et al., 2023), which may not be sufficient to form a nuanced opinion about the trustworthiness of AI in the health context. Future research should therefore take the degree of prior experience into account.

### Strengths and limitations

This study has several strengths, such as being preregistered, being sufficiently powered to reliably detect small effects, and having a sample that is representative of the German adult population with regard to gender, age, and education. Still, several limitations have to be acknowledged. All messages were generated by an AI chatbot and screened for high quality by a nutritional scientist. The high trustworthiness ratings therefore accurately reflect their quality. Although we deemed it important for ethical reasons not to knowingly circulate misinformation and to assign a source that did not author the messages, this limits the conclusions of this paper to high‐quality information, and larger trust effects could be plausible for low‐quality information and human‐generated messages with varying quality (Huschens et al., 2023). As such, the current study is only designed to evaluate the effects of AI labels, specifically, but not the detection and general quality AI health messages. Moreover, it could be speculated that at least some participants may make a difference between who wrote a text and underlying sources, which may have led to unintended interpretations of the source information below the text that specified that the text was written by ChatGPT. Furthermore, the study was conducted in an online panel, potentially limiting the generalizability of the findings. Although this may also mirror generally limited online message processing (Giese et al., 2020), the fact that more than 58% did not recognize the AI flag may also be seen as indication that the panel was not particularly paying attention to the survey, despite all participants having passed two attention checks. Finally, as chatbots become more widespread, their patterns of use may expand and diversify. Increased familiarity with AI tools and growing digital competence may foster greater acceptance of AI‐generated advice over time. Accordingly, user attitudes toward chatbot‐supported recommendations may become more favorable as experience, trust, and societal integration of these technologies increase (Horowitz et al., 2024).

### Conclusions

Source information is frequently overlooked, and even when it is recognized, it rarely influences how the statement is evaluated or how it is subsequently shared. The results point toward AI chatbots being an accepted and trusted source for nutrition‐related information in the population. The implications for public health messaging, especially a lack of differentiation by consumers between AI‐generated and expert‐generated content, need to be carefully considered by public health officials. Furthermore, there is a need for education and training regarding digital information and health literacy to mitigate potential adverse effects of incorrect online health information.

## CONFLICT OF INTEREST STATEMENT

The authors report that there are no competing interests to declare.

## ETHICS STATEMENT

General ethical approval was obtained from the University of Konstanz ethics committee (approval number 23/2021).

## Supporting information


**Table S1.** Socio‐demographic characteristics of the full sample and by condition.
**Table S2.** Results of independent samples t‐tests comparing ratings between AI and no source conditions.
**Table S3.** Results of between‐subjects ANOVAs comparing ratings between conditions.
**Table S4.** Results of the linear regression testing for moderating effects of prior experience with using AI to obtain nutrition‐related information and prior experience with using AI in general on trustworthiness for the full sample (*N* = 828).
**Table S5.** Results of between‐subjects ANOVAs comparing ratings between conditions, taking into account awareness of the AI source in the AI source conditions, in the full sample (*N* = 828).
**Table S6.** Results of the linear regression testing for the effects of the source and prior experience with AI on the willingness to share the information with others, taking into account awareness of the AI source in the AI source conditions. Analysis conducted with the full sample (*N* = 828).
**Table S7.** Results of logistic regression conducted with *N* = 415 participants in the AI conditions testing for effects of sociodemographic, psychological and behavioural characteristics on having correctly identified AI as the source (*n* = 141).

## Data Availability

Data and materials are available from the Open Science Framework: https://osf.io/t5arq/.
