# Supplementary material for: Perceptions of AI‐generated informational texts and effects of the source: An online experiment
Source: Appl Psychol Health Well Being. 2026 Oct 6;18(5):e70223. doi: 10.1111/aphw.70223 (PMC13641973; doi:10.1111/aphw.70223)
Supplement: Supplementary file 1 [file APHW-18-0-s001.docx]

# Perceptions of AI-generated informational texts and effects of the source: An online experiment

# Supplementary table

**Table S1.** Socio-demographic characteristics of the full sample and by condition.

|  |  | Full sample (*N* = 828) | By condition | | | | | |
| --- | --- | --- | --- | --- | --- | --- | --- | --- |
|  |  |  | AI: no  Topic: diets  (*n* = 140) | AI: no  Topic: supplements  (*n* = 137) | AI: no  Topic: snacks  (*n* = 136) | AI: yes  Topic: diets  (*n* = 136) | AI: yes  Topic: supplements  (*n* = 140) | AI: yes  Topic: snacks  (*n* = 139) |
| Age (M, SD) |  | 42.20, 13.44 | 42.85, 13.50 | 41.03, 13.50 | 42.20, 12.87 | 42.12, 14.30 | 42.50, 12.58 | 42.48, 14.00 |
| Gender^1^ | Women | 51.1% | 49.3% | 48.2% | 48.5% | 43.4% | 55.7% | 61.2% |
|  | Men | 48.9% | 50.7% | 51.8% | 51.6% | 56.6% | 44.3% | 38.8% |
| Residency | City with more than 100,000 inhabitants | 35.9% | 37.1% | 39.4% | 28.7% | 26.5% | 45.7% | 37.4% |
|  | City with between 20,000 and 100,000 inhabitants | 26.1% | 24.3% | 25.5% | 36.0% | 27.2% | 22.1% | 21.6% |
|  | Town with between 5,000 and 20,000 inhabitants | 18.7% | 20.7% | 15.3% | 14.7% | 21.3% | 16.4% | 23.7% |
|  | Village with up to 5,000 inhabitants | 19.3% | 17.9% | 19.7% | 20.6% | 25.0% | 15.7% | 17.3% |
| Years of education (M, SD) |  | 13.93, 3.12 | 13.94, 3.30 | 14.22, 3.21 | 13.45, 3.14 | 14.09, 3.22 | 14.09, 2.86 | 13.79, 2.98 |
| Monthly household budget |  | median = 2,500€ - 3,000€  interquartile range from 1,500€ - 2,000€ to 3,000€ - 5,000 € | median between 2,000€ - 2,5000€ and 2,500€ - 3,000€  interquartile range from 1,500€ - 2,000€ to 3,000€ - 5,000 € | median = 2,500€ - 3,000€  interquartile range from between 1,000€ - 1,500€ and 1,500€ - 2,000€ to 3,000€ - 5,000 € | median = 2,500€ - 3,000€  interquartile range from 2,000€ - 2,500€ to 3,000€ - 5,000 € | median = 2,500€ - 3,000€  interquartile range from 1,500€ - 2,000€ to 3,000€ - 5,000 € | median = 2,500€ - 3,000€  interquartile range from 1,500€ - 2,000€ to 3,000€ - 5,000 € | median = 2,500€ - 3,000€  interquartile range from 1,000€ - 1,500€ to 3,000€ - 5,000 € |
| Employment | full-time employment | 52.2% | 54.3% | 51.8% | 58.1% | 52.2% | 49.3% | 47.5% |
|  | part-time employment | 20.8% | 22.1% | 19.0% | 15.4% | 21.3% | 27.9% | 18.7% |
|  | job-seeking | 6.9% | 5.0% | 7.3% | 8.1% | 8.1% | 5.0% | 7.9% |
|  | in training | 5.9% | 4.3% | 8.0% | 5.9% | 6.6% | 2.9% | 7.9% |
|  | homemaker | 6.8% | 6.4% | 8.8% | 6.6% | 5.1% | 7.9% | 5.8% |
|  | retired | 7.5% | 7.9% | 5.1% | 5.9% | 6.6% | 7.1% | 12.2% |
| BMI (M, SD) |  | 27.02, 6.35 | 26.59, 5.93 | 26.90. 5.89 | 26.92, 6.18 | 26.91, 6.18 | 27.12, 6.55 | 27.63, 6.75 |
| Prior experience with AI for obtaining nutrition-related information | yes | 30.9% | 35.0% | 33.6% | 32.4% | 27.2% | 30.0% | 27.3% |
|  | no | 69.1% | 65.0% | 66.4% | 67.6% | 72.8% | 70.0% | 72.7% |
| Prior experience with AI in general (% yes) | yes | 36.7% | 34.3% | 39.4% | 36.0% | 30.9% | 35.0% | 44.6% |
|  | no | 63.3% | 65.7% | 60.6% | 64.0% | 69.1% | 65.0% | 55.4% |

*Note.* ^1^ No participant indicated diverse gender.

# Supplementary results

As preregistered, we conducted the same analyses with the full sample (*N* = 828; see Table S1), since more than 5% indicated that their data should not be used for research.

## Manipulation check

For the manipulation check, a chi-square test indicated that participants differed in indicating a source depending on the AI condition (AI vs no source). In the no source conditions, 93.0% of participants indicated that no source was provided, while only 60.2% in the AI conditions indicated that no source was provided, χ²(*df* = 1) = 123.66, *p* < .001, *Cramer V* = 0.39. The manipulation was thus unsuccessful in a substantial number of participants; we thus conducted an additional analysis taking source awareness into account (see below).

From the 165 participants in the AI condition who indicated that a source was provided, 141 participants had correctly identified AI, either by directly indicating ChatGPT (*n* = 85) or an AI more generally (*n* = 56). The remaining participants had given nonsensical responses (*n* = 10), indicated to not remember or to not know (*n* = 9), left the text box empty (*n* = 4) or indicated a university (*n* = 1).

## Are texts marked as produced by AI perceived differently compared to texts where no source is indicated?

First, we conducted independent samples t-tests to test for the effects of indicating that a text was produced by AI on trustworthiness, interest, perceived understanding, intention to act upon the information received, and willingness to share the received information with others. For trustworthiness, a very small effect was found, with the no source condition reporting somewhat higher trustworthiness (*M* = 5.34, *SD* = 1.33, 95% CI[5.21; 5.46]) than the AI source condition (*M* = 5.14, *SD* = 1.27, 95% CI[5.02; 5.27]). For the other four outcomes, p-values were above .05 (see Table S2). To test for potential differences between topics, we also conducted 2 *Source* x 3 *Topic* between-subjects ANOVAs with the same outcomes (see Table S3). The main effect on trustworthiness remained, *F*(1, 822) = 4.76, *p* = .029, *partial* η² = .01. There were no other main effects of Source (*p*s ≥ .197) nor interactions between Source and Topic (*p*s ≥ .055).

## Are the influences of AI moderated by prior experience with using AI to retrieve nutrition-related information or prior experience with using AI in general?

As pre-registered, we followed up the main effect for trustworthiness with a linear regression to for moderating effects of prior experience with AI (see Table S4). The main effect of AI source remained (*p* = .037), but there was no effect of prior experience with AI for either purpose (*p*s ≥ .633).

## Additional analyses taking source awareness into account

Since only a relatively small number of participants correctly identified an AI source in the AI source condition, we conducted additional 3 *Source Awareness (yes/ no/ no source provided)* x 3 *Topic* between-subjects ANOVAs to test whether source awareness might have influenced the results for the AI source conditions (see Table S5). In brief, there were no main effects or interactions for the outcomes trustworthiness, interest in the text, and intention (*p*s ≥ .085). For perceived understanding (*F*(2, 819) = 3.74, *p* = .024, *partial* η² = .01) and willingness to share (*F*(2, 819) = 3.67, *p* = .025, *partial* η² = .01), there were main effects of source awareness. Specifically, Bonferroni-corrected post-hoc tests revealed that participants in the AI conditions who were aware of the AI source found the text to be somewhat easier to understand (*M* = 5.84, *SD* = 1.34, 95% CI[5.58; 6.07]) than participants in the AI conditions who were unaware of the AI source (*M* = 5.41, *SD* = 1.49, 95% CI[5.24; 5.59]; *p* = .014). Furthermore, participants in the AI conditions who were aware of the AI source were somewhat less likely to share the information with others (*M* = 3.29, *SD* = 1.53, 95% CI[3.02; 3.56]) than participants in the AI conditions who were unaware of the AI source (*M* = 3.75, *SD* = 1.61, 95% CI[3.56; 3.95]; *p* = .018).

In line with the preregistration, we followed up the main effects with a linear regression, testing for potential moderation effects by prior experience with AI both in general and for obtaining nutrition-related information, for which p-values were greater than .05 (see Table S6).

## Do individuals explicitly mention if a text has been produced by AI when communicating about the information with others? Are certain sociodemographic, psychological or behavioural characteristics associated with the likelihood of mentioning AI as the source?

### Results based on the Whatsapp message item

A substantial number of participants refused to write a message (17.6%). Another 6.6% of participants left the box empty or used indicators such as “...” to signal that they would prefer not responding. Finally 5.0% of participants gave a nonsensical response. From the 586 participants (70.8% of the sample) who wrote a message, only 17 (i.e., 2.1%) mentioned AI as the message source. We therefore also did not conduct the planned analysis in the full sample.

### Results based on the manipulation check item

A logistic regression conducted amongst the participants in the AI conditions (*N* = 413, out of which *n* = 141 had correctly identified AI as the source; see also Table S7) indicated that the likelihood of correctly identifying AI as the source was higher in participants with more years of education (*p* = .020, OR = 1.11, 95%CI[1.01; 1.21]) and with higher nutrition knowledge (*p* < .001, OR = 1.16, 95%CI[1.08; 1.24]).

**Table S2.** Results of independent samples t-tests comparing ratings between AI and no source conditions.

| Outcome | AI condition (M, SD), *n* = 415 | No source condition (M, SD), *n* = 413 | *t* | *df* | *p* | Cohen’s *d* | | |
| --- | --- | --- | --- | --- | --- | --- | --- | --- |
|  |  |  |  |  |  | Point estimate | 95% CI lower bound | 95% CI upper bound |
| Trustworthiness | 5.14, 1.27 | 5.34, 1.33 | 2.17 | 826 | .030 | 0.15 | 0.02 | 0.29 |
| Interest | 4.60, 1.50 | 4.74, 1.64 | 1.29 | 826 | .197 | 0.09 | -0.05 | 0.23 |
| Perceived understanding | 5.55, 1.45 | 5.61, 1.51 | 0.59 | 826 | .555 | 0.04 | -0.10 | 0.18 |
| Intention | 4.32, 1.57 | 4.43, 1.66 | 0.99 | 826 | .162 | 0.07 | -0.07 | 0.21 |
| Willingness to share | 3.59, 1.59 | 3.59, 1.69 | -0.04 | 826 | .969 | -0.00 | -0.14 | 0.13 |

**Table S3.** Results of between-subjects ANOVAs comparing ratings between conditions.

|  | AI: no | | | AI: yes | | | AI source (*df*s 1, 822) | | | Topic (*df*s 2, 822) | | | AI source * topic (*df*s 2, 822) | | |
| --- | --- | --- | --- | --- | --- | --- | --- | --- | --- | --- | --- | --- | --- | --- | --- |
| Outcome | Topic: diets | Topic: supplements | Topic: snacks | Topic: diets | Topic: supplements | Topic: snacks | *F* | *p* | *partial* η² | *F* | *p* | *partial* η² | *F* | *p* | *partial* η² |
| Trustworthiness | 5.43 (1.25) | 5.34 (1.40) | 5.25 (1.33) | 4.94 (1.23) | 5.24 (1.25) | 5.25 (1.30) | 4.76 | .029 | .01 | 0.45 | .637 | < .01 | 2.88 | .057 | .01 |
| Interest | 4.94 (1.58) | 4.64 (1.71) | 4.63 (1.61) | 4.44 (1.48) | 4.75 (1.49) | 4.60 (1.54) | 1.67 | .197 | < .01 | 0.23 | .799 | < .01 | 2.91 | .055 | .01 |
| Perceived understanding | 5.61 (1.56) | 5.52 (1.59) | 5.71 (1.38) | 5.29 (1.48) | 5.57 (1.48) | 5.78 (1.37) | 0.38 | .540 | < .01 | 2.93 | .054 | .01 | 1.49 | .227 | < .01 |
| Intention | 4.36 (1.68) | 4.44 (1.75) | 4.48 (1-55) | 4.04 (1.57) | 4.46 (1.41) | 4.43 (1.69) | 1.02 | .312 | < .01 | 2.19 | .112 | .01 | 0.89 | .314 | < .01 |
| WIllingness to share | 3.65 (1.62) | 3.58 (1.71) | 3.52 (1.74) | 3.46 (1.53) | 3.80 (1.48) | 3.51 (1.75) | 0.00 | .975 | < .001 | 0.84 | .434 | < .01 | 1.11 | .332 | < .01 |

**Table S4.** Results of the linear regression testing for moderating effects of prior experience with using AI to obtain nutrition-related information and prior experience with using AI in general on trustworthiness for the full sample (*N* = 828).

| Predictor | *b* | SE(b) | 95% CI for b | | β | *t* | *p* |
| --- | --- | --- | --- | --- | --- | --- | --- |
|  |  |  | Lower bound | Upper bound |  |  |  |
| Constant | 5.26 | 0.09 | 5.08 | 5.43 |  | 59.08 | < .001 |
| AI source | -0.26 | 0.12 | -0.50 | -0.02 | -0.10 | -2.09 | .037 |
| Prior experience with AI to obtain nutrition-related information | -0.24 | 0.20 | -0.62 | 0.15 | -0.09 | -1.21 | .225 |
| Prior experience with AI in general | 0.33 | .019 | -0.04 | 0.69 | 0.13 | 1.77 | .077 |
| AI source * prior experience with AI to obtain nutrition-related information | 0.13 | 0.28 | -0.41 | 0.67 | 0.04 | 0.48 | .633 |
| AI source * prior experience with AI in general | 0.05 | 0.25 | -0.45 | 0.55 | 0.02 | 0.20 | .842 |

*Note.* AI source coding 0 = no source information, 1 = AI source. Prior experience with AI to obtain nutrition-related information coding 0 = no prior experience, 1 = prior experience; Prior experience with AI coding 0 = no prior experience, 1 = prior experience.

**Table S5.** Results of between-subjects ANOVAs comparing ratings between conditions, taking into account awareness of the AI source in the AI source conditions, in the full sample (*N* = 828).

|  | AI source (*df*s 2, 819) | | | Topic (*df*s 2, 819) | | | AI source * topic (*df*s 4, 819) | | |
| --- | --- | --- | --- | --- | --- | --- | --- | --- | --- |
| Outcome | *F* | *p* | *partial* η² | *F* | *p* | *partial* η² | *F* | *p* | *partial* η² |
| Trustworthiness | 2.47 | .085 | .01 | 1.28 | .279 | < .01 | 1.48 | .207 | .01 |
| Interest | 1.35 | .260 | < .01 | 0.48 | .616 | < .01 | 1.58 | .178 | .01 |
| Perceived understanding | 3.74 | .024 | .01 | .267 | .070 | .01 | .086 | .489 | < .01 |
| Intention | 1.93 | .146 | .01 | 3.30 | .037 | .01 | 0.61 | .655 | < .01 |
| Willingness to share | 3.67 | .025 | .01 | 1.36 | .258 | < .01 | 0.86 | .489 | < .01 |

**Table S6.** Results of the linear regression testing for the effects of the source and prior experience with AI on the willingness to share the information with others, taking into account awareness of the AI source in the AI source conditions. Analysis conducted with the full sample (*N* = 828).

| Predictor | *b* | SE(b) | 95% CI for b | | β | *t* | *p* |
| --- | --- | --- | --- | --- | --- | --- | --- |
|  |  |  | Lower bound | Upper bound |  |  |  |
| ***Perceived understanding*** |  |  |  |  |  |  |  |
| Constant | 5.55 | 0.10 | 5.35 | 5.75 |  | 54.81 | < .001 |
| AI source: no source (0) vs aware of indicated source (1) | 0.04 | 0.21 | -0.37 | 0.45 | 0.01 | 0.20 | .843 |
| AI source: no source (0) vs unaware of indicated source (1) | -0.17 | 0.16 | -0.47 | 0.13 | -0.05 | -1.10 | .272 |
| Prior experience with AI to obtain nutrition-related information | -0.26 | .22 | -0.70 | 0.18 | -0.08 | -1.16 | .245 |
| Prior experience with AI in general | 0.31 | 0.21 | -0.11 | 0.72 | 0.10 | 1.46 | .146 |
| AI source: no source (0) vs aware of indicated source (1) * prior experience with AI to obtain nutrition-related information | 0.37 | 0.41 | -0.43 | 1.16 | 0.05 | 0.90 | .369 |
| AI source: no source (0) vs unaware of indicated source (1) * prior experience with AI to obtain nutrition-related information | -0.40 | 0.37 | -1.13 | 0.32 | -0.08 | -1.10 | .273 |
| AI source: no source (0) vs aware of indicated source (1) * prior experience with AI in general | 0.10 | 0.37 | -0.62 | 0.83 | 0.02 | 0.28 | .783 |
| AI source: no source (0) vs unaware of indicated source (1) * prior experience with AI in general | 0.21 | 0.34 | -0.47 | 0.88 | 0.05 | 0.60 | .549 |
| ***Willingness to share*** |  |  |  |  |  |  |  |
| Constant | 3.26 | 0.11 | 3.04 | 3.47 |  | 29.65 | < .001 |
| AI source: no source (0) vs aware of indicated source (1) | -0.30 | 0.23 | -0.74 | 0.15 | -0.07 | -1.32 | .189 |
| AI source: no source (0) vs unaware of indicated source (1) | 0.21 | 0.17 | -0.12 | 0.54 | 0.06 | 1.25 | .211 |
| Prior experience with AI to obtain nutrition-related information | 0.42 | 0.24 | -0.06 | 0.89 | 0.12 | 1.73 | .085 |
| Prior experience with AI in general | 0.39 | 0.23 | -0.07 | 0.83 | 0.12 | 1.68 | .094 |
| AI source: no source (0) vs aware of indicated source (1) * prior experience with AI to obtain nutrition-related information | 0.49 | 0.44 | -0.38 | 1.35 | 0.06 | 1.10 | .270 |
| AI source: no source (0) vs unaware of indicated source (1) * prior experience with AI to obtain nutrition-related information | -0.02 | 0.40 | -0.81 | 0.76 | -0.00 | -0.06 | .953 |
| AI source: no source (0) vs aware of indicated source (1) * prior experience with AI in general | -0.24 | 0.40 | -1.02 | 0.55 | -0.4 | -0.60 | .551 |
| AI source: no source (0) vs unaware of indicated source (1) * prior experience with AI in general | 0.01 | 0.37 | -0.72 | 0.74 | 0.00 | 0.03 | .974 |

Note. Prior experience with AI to obtain nutrition-related information coding 0 = no prior experience, 1 = prior experience; Prior experience with AI coding 0 = no prior experience, 1 = prior experience.

**Table S7.** Results of logistic regression conducted with *N* = 415 participants in the AI conditions testing for effects of sociodemographic, psychological and behavioural characteristics on having correctly identified AI as the source (*n* = 141).

|  | *b* | SE(b) | Wald (*df* = 1) | *p* | OR | 95% CI for OR | |
| --- | --- | --- | --- | --- | --- | --- | --- |
|  |  |  |  |  |  | Lower bound | Upper bound |
| Constant | -4.00 | 1.00 | 16.18 | < .001 | 0.02 |  |  |
| Gender^a^ | 0.25 | 0.25 | 0.97 | .324 | 1.28 | 0.79 | 2.08 |
| Residency^b^ |  |  |  |  |  |  |  |
| *Medium-sized city* | -0.13 | 0.31 | 0.19 | .661 | 0.87 | 0.48 | 1.60 |
| *Small city* | 0.05 | 0.31 | 0.03 | .868 | 1.05 | 0.58 | 1.93 |
| *Village* | -0.04 | 0.32 | 0.02 | .893 | 0.96 | 0.51 | 1.79 |
| Income^c^ |  |  |  |  |  |  |  |
| *Second quartile* | 0.58 | 0.37 | 2.51 | .113 | 1.79 | 0.87 | 3.67 |
| *Third quartile* | 0.44 | 0.36 | 1.52 | .217 | 1.56 | 0.77 | 3.14 |
| *Fourth quartile* | 0.52 | 0.42 | 1.57 | .211 | 1.68 | 0.75 | 3.79 |
| Employment^d^ |  |  |  |  |  |  |  |
| *Part-time* | 0.08 | 0.30 | 4.51 | .789 | 1.08 | 0.60 | 1.95 |
| *Job-seeking* | 0.89 | 0.49 | 0.07 | .070 | 2.43 | 0.93 | 6.34 |
| *In training* | 0.48 | 0.51 | 3.29 | .348 | 1.62 | 0.59 | 4.40 |
| *Homemaker* | -0.16 | 0.55 | 0.88 | .767 | 0.85 | 0.29 | 2.50 |
| *Retired* | -0.05 | 0.48 | 0.01 | .914 | 0.95 | 0.37 | 2.42 |
| Age | 0.00 | 0.01 | 0.01 | .946 | 1.00 | 0.98 | 1.02 |
| Education | 0.10 | 0.04 | 5.44 | .020 | 1.11 | 0.12 | 1.21 |
| ATI | -0.04 | 0.11 | 0.16 | .688 | 0.96 | 0.78 | 1.18 |
| HEI | -0.05 | 0.03 | 2.11 | .146 | 0.96 | 0.90 | 1.02 |
| CoNKS | 0.15 | 0.04 | 18.19 | < .001 | 1.16 | 1.08 | 1.24 |

Notes. ^a^ comparator: women; ^b^ comparator: big city; ^c^ comparator: lowest quartile; ^d^ comparator: full-time employment
